# Extraction and Fractionation of Bioactives from *Dipsacus fullonum* L. Leaves and Evaluation of Their Anti-*Borrelia* Activity

**DOI:** 10.3390/ph15010087

**Published:** 2022-01-12

**Authors:** Piret Saar-Reismaa, Olga Bragina, Maria Kuhtinskaja, Indrek Reile, Pille-Riin Laanet, Maria Kulp, Merike Vaher

**Affiliations:** 1 Department of Chemistry and Biotechnology, School of Science, Tallinn University of Technology, 12618 Tallinn, Estonia; piret.saar1@taltech.ee (P.S.-R.); olga.bragina@taltech.ee (O.B.); maria.kuhtinskaja@taltech.ee (M.K.); pille-riin.laanet@taltech.ee (P.-R.L.); maria.kulp@taltech.ee (M.K.); 2 National Institute for Health Development, 11619 Tallinn, Estonia; 3 National Institute of Chemical Physics and Biophysics, 12618 Tallinn, Estonia; indrek.reile@kbfi.ee

**Keywords:** *Dipsacus fullonum* L., *Borrelia burgdorferi*, antibacterial activity, bis-iridoids, sylvestrosides, cytotoxicity

## Abstract

Lyme disease (LD) is a tick-borne bacterial disease that is caused by *Borrelia burgdorferi*. Although acute LD is treated with antibiotics, it can develop into relapsing chronic form caused by latent forms of *B. burgdorferi*. This leads to the search for phytochemicals against resistant LD. Therefore, this study aimed to evaluate the activity of *Dipsacus fullonum* L. leaves extract (DE) and its fractions against stationary phase *B. burgdorferi* in vitro. DE showed high activity against stationary phase *B. burgdorferi* (residual viability 19.8 ± 4.7%); however, it exhibited a noticeable cytotoxicity on NIH cells (viability 20.2 ± 5.2%). The iridoid-glycoside fraction showed a remarkable anti-*Borrelia* effect and reduced cytotoxicity. The iridoid-glycoside fraction was, therefore, further purified and showed to contain two main bioactives—sylvestrosides III and IV, that showed a considerable anti-*Borrelia* activity being the least toxic to murine fibroblast NIH/3T3 cells. Moreover, the concentration of sylvestrosides was about 15% of DE, endorsing the feasibility of purification of the compounds from *D. fullonum* L. leaves.

## 1. Introduction

Borreliosis, also known as Lyme disease (LD), is a well-known multisystemic bacterial disease. It is caused by *Borrelia burgdorferi* (*B. burgdorferi*), a spirochete, that is transmitted to humans from ticks. Lyme disease has various symptoms from localized skin deformities and joint pains, and even affects the heart and other muscles [1,2]. The most severe form is neuroborreliosis, which may lead to a variety of neurological manifestations from painful meningoradiculitis, ataxia, confusion and even ischemic stroke [3]. Modern medicine uses antibiotics to cure acute LD cases, where *B. burgdorferi* is mainly in an active spirochete form. When hostile conditions are introduced, for example, the widely used antibiotics such as doxycycline, amoxicillin and ceftriaxone [4,5], these bacteria can adopt different latent forms such as rounded bodies and aggregates (biofilm-like structures) that are considerably less affected by the treatment of one or even a mixture of antibiotics [5,6]. This ineffective treatment may lead to LD becoming persistent and/or resurfacing after being silent for a long time, named as a post-treatment Lyme disease syndrome that presents with chronic, persistent fatigue, musculoskeletal pain and neurocognitive difficulties for over 6 months after the primary treatment [7].

This leads to the search for novel substances that could be used as remedies. One possible source of new bacteriostatic phytochemicals is of natural origin—plants. The use of medicinal plants is widely known in case of various bacterial diseases. There are several reports demonstrating the ability of various bioactive plants to act as antibacterial agents. For example, grape (*Vitis vinifera*) seed extract [8], sugar leaf (*Stevia rebaudiana*) [9] and cat’s claw (*Uncaria tomentosa*) [6] have shown potential activity against acute *Borrelia* spirochetes and the nongrowing stationary phase. This is further supported by the use of treatments where phytochemicals are used in combination with antibiotics [10] or micronutrients such as vitamin D [11], enabling lower LD_50_ (median lethal dose) and MIC (minimum inhibitory concentration) concentrations against latent rounded forms of *B. burgdorferi*. This demonstrates a wide future potential of novel bioactives as both individual and synergic compounds for possible anti-*Borrelia* treatments [10,11].

**Figure 1:**
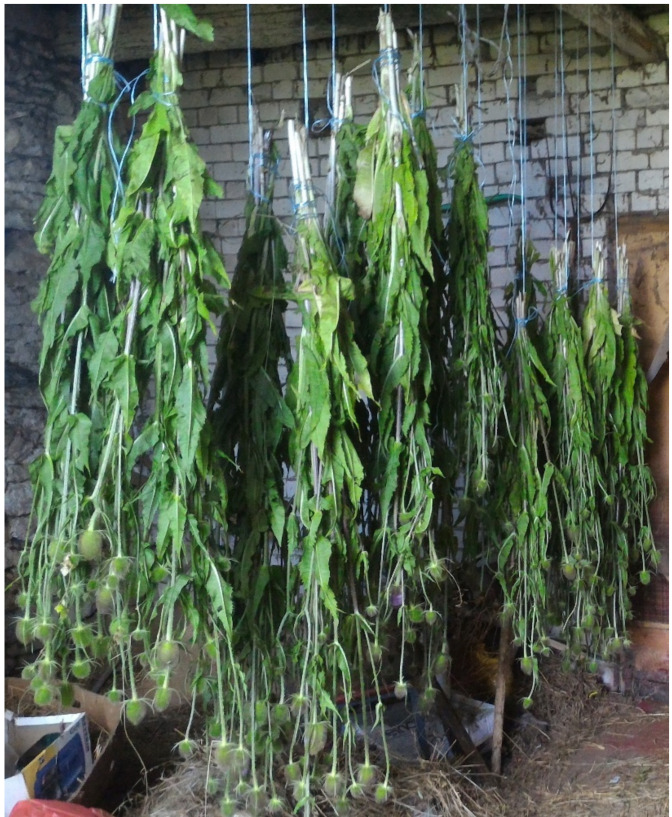
*Dipsacus fullonum* L. harvested from Saaremaa, Estonia, 2017.

A promising plant source is the genus *Dipsacus*. The root is known as “Xu duan” in Chinese medicine and is famous for its anti-cancer and anti-inflammatory properties and is even used for treating Alzheimer [12]. *Dipsacus* has mostly been applied as an arthritis remedy [13]. One of the most studied species is *D. asper*, with the focus on the root or the seeds of the plant [12]. *Dipsacus fullonum* L., also known as wild teasel (shown in Figure 1) and its roots have also been characterized as a potential source of anti-*Borrelia* bioactives [14]. Previously, all the known anti-*Borrelia* research focused on the radix—the root of the plant with minimal activity against Lyme disease [15]. It has also been demonstrated that the composition of the leaves significantly differs from that of the root [16], demonstrating good antioxidant and antimicrobial activities [16,17]. Moreover, our research group’s investigations have established the in vivo effectiveness of teasel leaves extracts against several cancer strains [18].

The current study aimed to continue our research group’s earlier investigations on *D. fullonum* L. leaves [18,19] by elucidating, fractionating, identifying and quantifying the content of *D. fullonum* L. bioactives (iridoids and polyphenolics) to evaluate their anti-*Borrelia* activity against stationary phase *Borrelia burgdorferi*. Additionally, the composition of the more promising fractions was identified and quantified together with their antioxidative capacity, anti-*Borrelia* activity and cytotoxicity evaluated.

## 2. Results and Discussion

**Figure 2:**
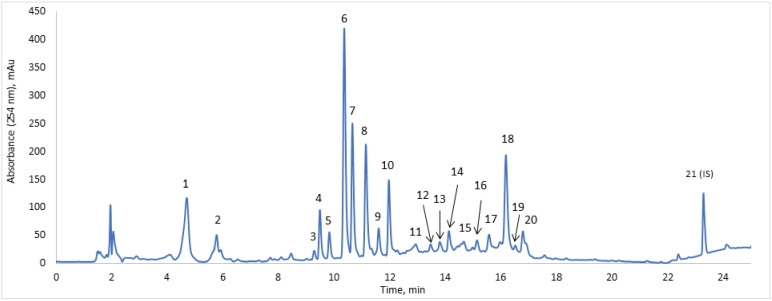
Chromatogram of DE, with selected peaks for quantification. The results of identification are presented in Table 1. Peak 21—internal standard, bicalutamide, 40.0 mg/L.

Recently, the extract from *D. fullonum* L. leaves was evaluated; however, the analysis was limited to the antioxidant, anticholinesterase and antidiabetic effects only [16]. To evaluate the leaf extract for anti-*Borrelia* effects, the first step was the analysis of DE by HPLC–DAD–MS to obtain a reliable chromatographic separation for the determination and quantification of the main constituents. A total of 20 major components were identified and quantified in the extract. A representative chromatogram of DE is shown in Figure 2. DE was diluted 20 times for evaluation against stationary phase *B. burgdorferi* with a gross concentration of 305.5 ± 24.5 mg/L of the chosen 20 compounds of interest. The quantification was achieved as described in the previous section according to the DAD signal and chlorogenic acid, loganin, saponarin, isoorientin or isovitexin calibrations, accordingly.

The identification was performed using available reference standards and literature comparison to the MS/MS fragmentation pattern and UV spectra. Two main classes of substances were identified: polyphenols and iridoids. The two major polyphenols were saponarin (apigenin-6-C-glucoside-7-O-glucoside, peak 6) and chlorogenic acid (peak 1), which were the most abundantly found species in previous studies of *D. fullonum* L. leaves [16] and *D. sativus* leaves [17]. The main iridoids were sylvestrosides III and IV (peak 18), and loganic acid and loganin (peaks 2 and 4, respectively).

Quantitatively, the highest amounts of compounds were as follows: saponarin (17.2%), chlorogenic acid (17.1%), the mixture of sylvestrosides III and IV (15.2%) at a ratio of 0.9:1.0, dichlorogenic acid isomer (14.2%), isoorientin (5.8%), loganin (4.9%) and isovitexin (4.2%), correspondingly. Although orientin [16] and isoorientin [17] have both been reported in literature, the MS^2^ fragmentation pattern and comparison to the chromatographic parameters of the orientin standard clearly demonstrated peak 7 to be isoorientin instead of orientin. The concentrations of the tested DE on the stationary phase of *Borrelia* with identification parameters are given in Table 1.

Using characteristic peaks from each NP fraction, dilutions were made to correlate gross concentrations with that of DE. All the diluted NP fractions were then used for primary screening against stationary phase *Borrelia burgdorferi* in vivo to detect active fractions for further analysis. The results from the primary evaluation are given in Table 2, where the fraction was considered active if it revealed a significant effect on the residual viability of the stationary phase *B. burgdorferi*.

The first normal phase fraction, NP1, did not contain any of the raw extract compounds at 254 nm, which could be explained by the fact that NP1 contained mainly chlorophylls that were not soluble in 70% EtOH and did not absorb. This was further correlated by the TLC results, where the first fractions had a characteristic emission at 312 nm corresponding to chlorophylls [20]. No activity was observed in fraction NP3, which included loganin and loganic acid ethyl ester, and was first purified from *D. asper* roots and showed moderate neuroprotective effects [21]. Loganin has been frequently identified in various *Dipsacaceae* plants in roots, seeds and leaves [12]. Similarly, fraction NP4 contained mainly loganin derivatives, which did not show any specific activity against the tested cultures either. The lack of anti-*Borrelia* activity could be explained by the low concentration of the compounds.

However, several of the fractions demonstrated in vitro activity against stationary phase *B. burgdorferi* in the concentration ranges similar to those of raw extracts. The main constituents of NP2 were assigned to iridoid glycosides, having *m*/*z* values of 583 and 585. A similar fraction produced and tested previously by our group has shown activity against the selective cytotoxic effect on human breast cancer cell lines MCF7 and MDB-MD-231 [18]. Fraction NP5 contained mainly loganic acid, which also exhibited anti-*Borrelia* activity, similarly to iridoids from fraction NP2. Moreover, loganic acid has been shown to have anti-infectivity activity [22] and thus fractions NP2 and NP5 were further analyzed.

Fractions NP6 to NP8 contained various phenolic compounds, mainly glycosides of apigenin and luteolin, as well as chlorogenic acid and its derivates. Although flavonoids such as apigenin and luteolin have shown to be cytotoxic, their glycosides were much less so [23]. Previously, the phenolic extracts from *D. sativus* containing saponarin, isoorientin and isovitexin have been proven to reveal antioxidant activities [17], and moreover, saponarin has been recently shown to possess anti-inflammatory and anti-allergic effects [24]. The other main constituents were chlorogenic acid (NP8) and dichlorogenic acid isomers (NP7). As chlorogenic acid has shown potential anti-cancer and other health-beneficial effects, but in much higher concentrations (2 mM or above), it may also have cytotoxic effects [25], fraction NP8 was discarded from additional experiments and only NP7 was used as it contained similar substances. To further evaluate the possible synergistic effects of individual substances, isovitexin and luteolin were used as standards. Isovitexin showed cytotoxicity at active concentrations or even three times diluted (100 mg/L) (data not shown); thus fraction NP6 was also excluded from further specific analysis.

As a result, the 70% EtOH (*v*/*v* %) DE, NP2, NP5 and NP7 remained as possible candidates for additional in-depth analysis with stationary phase *B. burgdorferi*. To further evaluate the possible synergistic effects of the fractions under analysis a designed mixture (DM) composed of NP2 and NP7 fractions at a ratio of 1:3 was made to replicate the iridoid and polyphenol fractions without additional substances. All the viability results were compared to those of a mixture of the most common antibiotics for the early stage Lyme borreliosis doxycycline and cefuroxime [2]. The residual viability of stationary phase *B. burgdorferi* of samples is given in Table 3.

The raw DE has the highest anti-*Borrelia* activity, 19.8 ± 4.7%, followed by NP5 (loganic acid) and NP7 (saponarin, isoorientin and dichlorogenic acid derivatives), all below 30%. Previously, only the lipophilic extract of *D. fullonum* L. has shown anti-borrelia effects [14], but the results of this study show that a hydrophilic extract contains several anti-*Borrelia* compounds. The iridoid fraction NP2 and the DM mixture demonstrated moderate effects against the stationary phase bacteria. A recent study reported that the combination of doxycycline, cefoperazone and daptomycin successfully eliminated both spirochetes and persisters [26]. Our data from the present study also confirmed the effectiveness of this triple-antibiotic combination on *B. burgdorferi* by significantly reducing the viability of the stationary phase culture, by about 76%.

As polyphenols have been previously extensively researched and have shown significant anti-oxidative and anti-cancer effects [27] as well as relatively high cytotoxicity [28], the focus was on fraction NP2, which contained iridoid glycosides. As the HPLC–DAD–MS analysis demonstrated that this fraction contained several bis-iridoids as well as sylvestrosides III and IV, further purification was performed by RPFC as described in the Methods and Materials section. The NP2-RP fraction obtained was reanalyzed by HPLC–DAD–MS and was further confirmed by NMR analysis to contain sylvestrosides III and IV at a ratio of 0.9:1:0 with slightly more sylvestroside IV, as described in Section 3.4. Previously, both sylvestroside III and IV have been found in *Dipsacus laciniatus*, where sylvestroside IV was in abundance compared to sylvestroside III, 5.2 and 0.8 mg in 100 g fresh plant, respectively [29]. In *D. fullonum* L., the ratio has also been with mostly sylvestroside IV (153 mg/100 g fresh material) to sylvestroside III (100 mg/100 g fresh material) [30]. The anti-inflammatory activity of sylvestrosides I, II, III and IV have been previously demonstrated by Chen et al. [31] contributing to the potential for investigation as anti-*Borrelia* compounds. Therefore, NP2-RP was subjected to in vitro testing against stationary phase *B. burgdorferi* with positive results as the fraction provided a residual viability of 64.5 ± 14.0%. Moreover, sylvestroside III and IV were in abundance in the overall extract at 15.2% of gross concentration, which is similar to previously published amount of 10% of sylvestroside III by Oszmiański et al. [16]. Although they identify only sylvestroside III, it is assumed that the combination of sylvestroside III and IV is meant as the use of HPLC–DAD–MS with water–ACN as eluents does not provide separation of III and IV. Therefore, the bioactives from the iridoid fraction NP2 were sylvestrosides III and IV and could be further tested for additional anti-*Borrelia* activity as separate bioactives or in combination with other phytochemicals/antibiotics.

**Figure 3:**
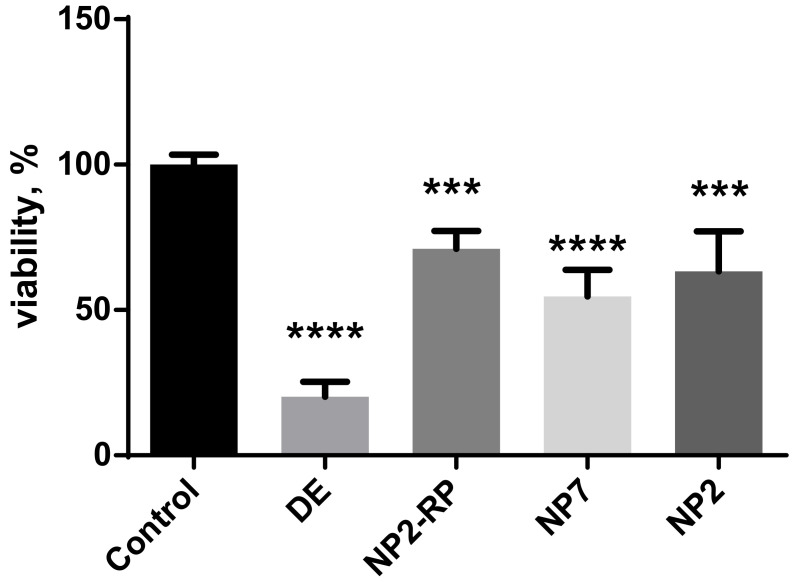
Cytotoxic effect of DE and its fractions on NIH 3T3 cells assessed by WST1 assay after 48 h. The experiments were run triplicate. The graphs represent as mean ± SD. Cells treated with 60% EtOH were normalized to 100% as control; *p* < 0.001 as ***, *p* < 0.0001 as ****.

The evaluation of DE and its fractions also included the determination of cytotoxicity against normal mammalian cells to ensure the applicability of the compounds in the used concentrations. The cytotoxicity of DE and its fractions was determined using the WST-1 assay, where NIH 3T3 cells were treated with DE and its fractions and the viability parameters were registered after 48 h and are presented in Figure 3. The results obtained clearly showed the need for fractionation and purification of the raw extract, which had only a 20.2 ± 5.2% cell viability, against the 71.1 ± 6.1% of the NP2-RP fraction. Based on the WST-1 assay results, the cytotoxicity arose from polyphenols as the NP7 fraction had a viability of 54.7 ± 9.2%. These results correlate with those of our previous studies on iridoid fractions having a 63.3 ± 13.7% viability [18]. The detailed results of the statistical comparison test are given in Supplementary Materials File S1.

Moreover, the antioxidant activity of the active fractions was evaluated by the oxygen radical absorbance capacity. The raw extract demonstrated an activity of 10.8 ± 0.8 mmol TE/100 mL, being similar to the previously obtained results for *D. fullonum* L. leaves extract [16]. NP7 exhibited the highest activity, 12.5 ± 0.1 mmol TE/100 mL, which corresponds to that of the polyphenolic fraction, including flavonoids saponarin and isoorientin. These flavonoids are well known as antioxidants and free-radical scavengers [17]. The iridoid-glycoside fractions NP2 and NP2-RP tested only showed an activity of 0.78 ± 0.06 and 0.27 ± 0.02 mmol TE/100 mL, respectively. The results correlate with the data of previous studies on the ORAC of *D. fullonum* L. leaves extract by Oszmianski et al. (2020) [16] and further confirms their proposed activity, originating from polyphenolics rather than iridoids.

## 3. Materials and Methods

### 3.1. Plant Material

The *Dipsacus fullonum* L. was collected from Saaremaa, Estonia, in the summer of 2017 (pictured in Figure 1). The identification of the plant was performed by comparison with an authentic specimen *Dipsacus fullonum* L., and a voucher specimen was deposited in the Herbarium of the Institute of Agricultural and Environmental Sciences of the Estonian University of Life Sciences, herbarium specimens TAA0153271–TAA0153274. The leaves were separated manually, washed with Milli-Q water and dried in a well-ventilated room at room temperature (25–30 °C) for four weeks. The dried leaves were powdered in a mechanical Fritsch Universal Cutting Mill pulverisette 19 grinder (Weimar, Germany) and mixed together. The mixture was stored in an air-tight container protected from sunlight in the laboratory at 23 °C.

### 3.2. Preparation of D. fullonum Extract

The extraction procedure was modified from our previous study [19]. Briefly, 50.0 g of the pulverized leaves of *D. fullonum* L. were weighed into a flask and 250 mL of 70% (*v*/*v* %) ethanol (EtOH) was added. The extract was shaken on an Orbital Shaker DOS-20M (Elmi, Newbury Park, CA, USA) for 25 min at 250 rpm and ultrasonicated at 40 °C for 30 min. The extract was decanted into a volume cylinder. Another 250 mL of 70% EtOH was added and shaken for 30 min and the extracts were combined. The raw *D. fullonum* L. leaves extract (DE) was then filtered through a blue Whatman filter paper (Buckinghamshire, UK) and used for further extraction or analysis.

### 3.3. Isolation of D. fullonum L. Constituents

The *D. fullonum* L. leaves extract was concentrated 20 times by volume, using a rotary Laborota 4000, Heidolph (Schwabach, Germany) evaporator. Eight milliliters of the concentrated extract was added to 7 g of silica gel. The extract was rotated dry on a rotary evaporator and subjected to normal-phase silica gel column (40–100 µm) (40 mm, 140 mm) (60 g, CHCl_3_ → CHCl_3_:MeOH 9:1 → CHCl_3_:MeOH 8:2 → CHCl_3_:MeOH 7:3 → CHCl_3_:MeOH 6:4 → CHCl_3_:MeOH 5:5 → CHCl_3_:MeOH 4:6 → CHCl_3_:MeOH 3:7 → CHCl_3_:MeOH 2:8 → CHCl_3_:MeOH 1:9 → MeOH) to give 30 fractions per column. Altogether, 16 columns were made. The collected normal-phase fractions were analyzed using thin-layer chromatography (TLC) to combine similar fractions. Three microliters of each fraction was manually applied to 5 × 10 cm TLC silica gel 60 F254 (0.20 mm) sheets (Macherey-Nagel, Düren, Germany). The sheets were placed in a saturated development chamber with a reagent mixture of n-butanol:Milli-Q water:EtOH 4:1:1. The plates were air-dried and subjected to analysis at 254 and 312 nm. Accordingly, the first 30 fractions were combined into 8 normal-phase fractions (NP), NP1 to NP8 corresponding to the TLC bands. NP1 to NP8 were evaporated dry and resolubilized in 70% EtOH. All eight NP fractions were then analyzed using HPLC–DAD–MS.

Fraction NP2 was further subjected to reversed-phase flash chromatography (RPFC) for further purification. The purification was achieved by elution through the Biotage Sfär C18 column (12 g, 30 µm, 100 Å, Uppsala, Sweden). Elution in RPFC was carried out with a mobile phase of 20% ACN/80% Milli-Q water (isocratic elution) with a flow rate of 30 mL/min. The elutes were scanned continuously at 210 and 238 nm throughout the experiment. The fractions were automatically collected into the test tubes. Each fraction (without any additional evaporation) was subjected to the HPLC–DAD–MS analysis. The fractions with a high iridoid content and maximally free of contaminants were combined and evaporated to dryness giving NP2-RP as yellow-brownish crystals, which were analyzed by HPLC–DAD–MS and NMR spectroscopy.

### 3.4. NMR Analysis

Nuclear magnetic resonance analysis was carried out on a 500 MHz Agilent DD2 spectrometer equipped with a 5 mm ID probe. Fifty milligrams of the product fraction NP2-RP was dissolved in 600 µL of acetone-d_6_ and subjected to analysis at 25 °C sample temperature. Spectra were referenced according to the residual solvent peak at 2.05 ppm. Identification of products was achieved by conducting ^1^H, ^13^C, COSY, TOCSY, HSQC, HMBC and H2BC analysis of the mixture and comparing spectra with literature data [29,30,32]. Several ^1^H signals of the mixture components were overlapping in 1D spectra and were resolved only by 2D methods, making the identification of multiplet structures complicated. Sylvestroside III and sylvestroside IV were identified as the major components of NP2-RP with a molar ratio of 0.9:1.0. The ratio was established by comparing HSQC signal integrals from sylvestroside III and sylvestroside IV iridoid unit’s bridging 5- and 9-positions. Assuming the relaxation and C-H coupling parameters of both positions in either compound are likely similar, HSQC integrals allow establishing the relative abundance of both. Integral comparisons for 5- and 9-positions in either compound gave the same result. Full NMR spectra are shown in Supplementary Materials File S2. The compounds were characterized as:

Sylvestroside III: ^1^H-NMR (500 MHz, acetone-d_6_) δ = 9.71 (broad, 1H), 7.59 (broad, 1H), 7.55 (broad, 1H), 5.62 (m, 1H), 5.47 (m, 1H), 5.26 (m, 1H), 5.21 (m, 1H), 5.00 (d, J = 6.2, 1H), 4.72 (m, 1H), 3.87 (d, J = 12.1 Hz, 1H), 3.67 (d, 9.5 Hz, 1H), 3.65 (s, 3H), 3.44 (m, 2H), 3.43 (m, 1H), 3.38 (m, 1H), 3.25 (m, 1H), 3.06 (m, 1H), 2.78 (m, 1H), 2.74 (m, 1H), 2.48 (m, 1H), 2.29 (m, 1H), 2.13 (m, 1H), 1.92 (m, 1H), 1.65 (m, 1H), 1.06 (d, J = 7.1 Hz, 3H); ^13^C-NMR (125 MHz, acetone-d_6_) δ = 206.4, 168.1, 166.8, 153.3, 153.0, 134.8, 120.2, 111.5, 109.7, 99.7, 96.7, 96.5, 77.9, 77.7, 77.2, 74.3, 71.3, 62.7, 51.3, 48.0, 44.8, 44.7, 41.2, 40.3, 33.2, 27.1, 14.3.

Sylvestroside IV: ^1^H-NMR (500 MHz, acetone-d_6_) δ = 9.7 (broad s, 1H), 7.53 (broad, 1H), 5.6 (m, 1H), 5.48 (m, 1H), 5.23 (m, 3H), 4.72 (m, 1H), 4.42 (dd, J = 11.6 Hz, 5.6 Hz; 1H), 4.22 (m, 1H), 3.87 (d, J = 12.1 Hz, 1H), 3.73 (s, 3H), 3.69 (broad, 1H), 3.67 (d, 9.5 Hz, 1H), 3.44 (m, 1H), 3.43 (m, 1H), 3.38 (m, 1H), 3.37 (m, 1H), 3.25 (m, 1H), 3.07 (m, 1H), 2.79 (m, 1H), 2.48 (dd, J = 17.5 Hz, 7.5 Hz, 1H), 2.40 (m, 1H), 2.74 (m, 1H), 2.14 (dd, J = 13.5 Hz, 7.0 Hz; 1H), 2.08 (broad, 1H), 1.65 (m, 2H), 1.63 (m, 1H), 1.02 (d, J = 6.6 Hz, 1H); ^13^C-NMR (125 MHz, acetone-d_6_) δ = 201.3, 169.8, 169.5, 166.8, 153.3, 134.8, 120.2, 109.9, 99.7, 96.8, 79.2, 77.9, 77.7, 74.3, 71.3, 70.0, 62.8, 52.8, 52.1, 44.9, 44.8, 42.7, 41.9, 38.8, 37.3, 27.2, 13.5.

### 3.5. HPLC–DAD–MS Analysis

The HPLC–DAD–MS 1200 Series HPLC instrument (Agilent Technologies, Inc., Palo Alto, CA, USA) system equipped with an Agilent Poroshell 120 EC-C18 column (2.7 µm particles, 4.6 × 100 mm, Santa Clara, CA, USA) was used for the analysis of DE and its fractions. The gradient elution used 7 mM ammonium acetate solution for solvents A and ACN with 0.1% formic acid for solvent B. The gradient for solvent A was 0 min 95%, 10 min 75%, 18.5 min 62.3%, 21 min 5%, 26 min 5%, 26.01 min 95%, 31 min 95%. The flow rate was 0.6 mL/min and the injection volume was 5 µL. The DAD spectra were registered from 200 to 400 nm. The parameters for MS analysis were set in the negative ion mode, with the spectra obtained in the m/z range from 100 to 1000. Nitrogen was used as the nebulizing and drying gas, and helium served as the collision gas. Peak identification was achieved by MS/MS analysis.

The quantification of the main substances was performed by HPLC–DAD according to the relative absorbance signal (in relation to the internal standard—bicalutamide, 40 mg/L) at 254 nm according to the calibration curves of standards (chlorogenic acid, loganin, orientin, saponarin and isovitexin). Loganin was expressed as loganin, but the other iridoids (loganic acid, loganic acid ethyl ester, all bis-iridoid glycosides and silvestrosides III and IV) were calculated as loganin compounds. Chlorogenic acid and dichlorogenic acid isomers were expressed as chlorogenic acid compounds. Saponarin and luteolin derivates were calculated as saponarin compounds, isoorientin as an orientin compound and isovitexin directly from the relative standard curve.

### 3.6. ORAC Assay

The biological activity of DE and the prepared fractions was measured using the oxygen radical absorbance capacity (ORAC) with minor modifications as described by Naguib [33]. The fresh stock solutions were made each day and composed of 100 mM phosphate buffer (pH = 7.4), 10 nM fluorescein in buffer, 300 µM 6-hydroxy-2,5,7,8-tetramethylchroman-2-carboxylic acid (Trolox) in buffer and 600 mM 2,2′-azobis (2-amidino-propane) dihydrochloride (AAPH) in buffer.

The total volume of the reaction mixture was 3 mL, the mixture was composed of 2.7 mL of 10 nM fluorescein and 100 µL sample/Trolox dilution, which was incubated at 37 °C for 10 min, and then 200 µL of 40 mM AAPH was added. The samples were measured by a Hitachi F-7000 Fluorescence Spectrophotometer (Chiyoda, Tokyo, Japan) at λ_ex_/_em_ 495/520 nm, slits 5 nm, and the time scan was recorded for 1800 s once per s. The calibration for a well-known antioxidant, Trolox, was measured from 1 to 10 µM and was given as area under curve change from 0 µM Trolox (blank sample). All samples were measured in triplicate and the results given in mean Trolox equivalents per 100 mL of DE (mM TE/100 mL) ± standard deviation (SD).

### 3.7. Bacterial Strain, Media and Culture

Low passage isolates (≤8) of *Borrelia burgdorferi* strain B31 were obtained from the American Type Culture Collection (Manassas, VA, USA). *Borrelia burgdorferi* was cultured in BSK-H medium with 6% rabbit serum. All culture media were filter-sterilized using a 0.2 µm filter. The cultures were incubated in 50 mL sterile closed conical tubes at 33 °C in 5% CO_2_ without antibiotics. After incubation for 7 days, the *B. burgdorferi* culture went into stationary phase (~10^7^ spirochetes/mL) [9,34], followed by the transferring of the bacterial cultures into 96-well tissue culture microplates for fraction screening.

### 3.8. SYBR Green I/PI Assay

To estimate the viability of *B. burgdorferi*, the SYBR Green I/propidium iodide (PI) assay was performed as described by Feng et al. [35]. Briefly, SYBR Green I 5 µL (100 × stock, Invitrogen, Waltham, MA, USA) and 5 µL propidium iodide (0.5 mM, Sigma, St. Louis, MO, USA) were added to each well and mixed thoroughly. The plates were incubated in the dark for 15 min at room temperature, with λ_ex_ at 450 nm and λ_em_ at 535 nm (green emission) and 635 nm (red emission) for each well of the screening plate using a TECAN Genios Pro microplate reader (Männedorf, Switzerland). Meanwhile, *B. burgdorferi* suspensions (live and 70% isopropyl alcohol killed) at five different proportions of live: dead cells (0:10, 2:8, 5:5, 8:2, 10:0) were mixed and added to the wells of the 96-well plate. Then, the SYBR Green I/PI reagent was added to each of the five samples, and the green/red fluorescence ratios for each proportion of live/dead *B. burgdorferi* were measured using the same parameters on the TECAN Genios Pro microplate reader as above. Using least-square fitting analysis, the regression equation and regression curve of the relationship between the percentage of live bacteria and the ratios of green/red fluorescence were obtained. The regression equation was used to calculate the percentage of live cells in each well of the screening plate.

### 3.9. Evaluation of Bactericidal Effect of Test Compounds

To evaluate the possible activity of DE and its fractions against stationary phase *B. burgdorferi*, the probes were added to 100 µL of the seven-day old *B. burgdorferi* culture in the 96-well plate. The entire experiment was repeated in triplicate for each concentration of the tested compounds. All plates were incubated at 33 °C in 5% CO_2_ for the next 7 days. Control cultures were treated with doxycycline, daptomycin and cefoperazone with a final concentration 50 µM. The live and dead cells were evaluated using the SYBR Green I/PI assay and the viability % was calculated through the regression equation.

### 3.10. Cell Culture

The murine fibroblast NIH/3T3 cell line was obtained from the American Type Culture Collection. The cells were propagated in Dulbecco’s modified Eagle’s medium (DMEM) (Gibco, Waltham, MA, USA) supplemented with 10% bovine calf serum (Gibco) and 5% penicillin/streptomycin. The cells were incubated at 37 °C in a humidified 5% CO_2_ and 95% air atmosphere.

### 3.11. Treatment Procedure and Sample Preparation

The cells were plated at a density of 2.5 × 10^5^ cells/well (The Countess^®^ Automated Cell Counter, Invitrogen) in 96-well plates and incubated overnight. After 24 h of incubation, 100 μL of fresh media or fresh media containing the 70% EtOH (*v*/*v* %) DE, NP2, NP5, NP7 and NP-RP (up to 5 μL of each probe was added to 100 μL fresh media) were added, and the cells were incubated for an additional 48 h.

### 3.12. Cell Viability Measured by WST-1

The effect on the viability of cells was determined using the cell viability assay WST-1 (Roche Applied Science, Penzberg, Germany). WST-1 allows colorimetric measurement of cell viability due to reduction of tetrazolium salts to water-soluble formazan by viable cells. The amount of the formed formazan dye correlates with the number of viable cells. The measurements were completed 48 h after treatment of the cells. The experiments with addition of 5 μL of 96% EtOH were used as a control. Five microliters per well of WST-1 reagent was added to 100 µL cell culture medium, incubated at 37 °C for 2 h and absorbance was measured at 450 nm using the TECAN Genios Pro microplate reader.

### 3.13. Statistical Analysis

Statistical analysis was performed by using one-way analysis of variance (ANOVA) with the post hoc Dunnett’s multiple comparison test. All measurements represent data from at least three independent experiments, all performed in triplicate as mean ± standard deviation (SD). The optical density of the control—cells treated only with solvent—was taken as 100% viability. Statistical significance of *p* < 0.05 is represented as *, *p* < 0.001 as ***, *p* < 0.0001 as ****. Statistical analyses were performed with GraphPad Prism 7.

## 4. Conclusions

The ethanolic extract from *Dipsacus fullonum* L. leaves shows great potential as a possible source for novel lead compounds against stationary phase *Borrelia burgdorferi*. Although DE demonstrates the highest anti-*Borrelia* activity, the cytotoxicity of the raw extract clearly demonstrates the need for isolation of the most active and less cytotoxic fractions. The main compound classes from DE were polyphenols such as saponarin, isoorientin and isovitexin, and iridoids such as loganin, loganic acid and sylvestrosides III and IV. The highest cytotoxicity was found to originate from polyphenols, but was overcome by fractionation and purification to enhance the positive effects and reduce potential cytotoxic additives. The main active bis-iridoid compounds were fractionated, purified and identified as sylvestrosides III and IV by HPLC–DAD–MS/MS and verified by NMR. The bioactive sylvestrosides have great potential as noncytotoxic compounds for further testing against the latent forms of *B. burgdorferi* as separate phytochemicals or in combination with other bioactives, antibiotics and micronutrients.

## Figures and Tables

**Table 1:** Identification and concentrations of DE compounds.

Peak	Compound	T_ret_, Min	λmax, nm	MS, *m*/*z*	MS/MS, *m*/*z*	Conc., mg/L
1	Chlorogenic acid	4.7	245; 325	353	191	47.8 ± 2.7
2	Loganic acid	5.8	230	375	213; 169	14.6 ± 2.1
3	Luteolin derivative	9.3	270; 350	609	447; 325	0.8 ± 0.3
4	Loganin	9.5	238	389	227; 209	13.6 ± 0.7
5	Loganic acid ethyl ester	9.8	245	403	395; 357	6.0 ± 0.5
6	Saponarin	10.4	270; 335	593	311; 431; 473	48.1 ± 6.1
7	Isoorientin	10.7	347	447	557; 327; 429	16.3 ± 2.1
8	Dichlorogenic acid isomer	11.1	325	515	353; 191	39.7 ± 1.9
9	Dichlorogenic acid isomer	11.6	325	515	353; 173; 203	3.6 ± 3.2
10	Isovitexin	12.0	268; 340	431	311; 341; 413	11.7 ± 0.5
11	bis-Iridoid glycoside	12.9	238	541	–	4.4 ± 0.8
12	bis-Iridoid glycoside	13.5	240	585	373	0.8 ± 0.3
13	bis-Iridoid glycoside	13.8	240	583	513; 459	1.0 ± 0.6
14	bis-Iridoid glycoside	14.1	240	585	373	5.0 ± 0.7
15	bis-Iridoid glycoside	14.7	240	585	373	4.8 ± 0.9
16	bis-Iridoid glycoside	15.1	240	585	373	2.9 ± 0.4
17	bis-Iridoid glycoside	15.6	240	583	373	4.9 ± 1.6
18	Sylvestrosides III and IV	16.2	240	583	373	42.5 ± 4.7
19	bis-Iridoid glycoside	16.5	240	583	373	0.9 ± 0.2
20	bis-Iridoid glycoside	16.8	240	583	373	10.5 ± 0.1
21	Bicalutamide (IS)	23.3	215; 270	429	255; 183	500

**Table 2:** Preliminary screening for activity against stationary phase *B. burgdorferi*.

NP Number	Main Constituents	Activity
NP1	Chlorophylls	−
NP2	bis-Iridoids (*m*/*z* 585, 583)	+
NP3	Iridoids—loganin, loganic acid ethyl ester	−
NP4	Loganin derivatives	−
NP5	Loganic acid	+
NP6	Isovitexin, saponarin, isoorientin	+
NP7	Saponarin, isoorientin, 2 chlorogenic acid derivatives	+
NP8	Saponarin, isoorientin, chlorogenic acid	+

**Table 3:** Effect of DE and its fractions on the seven-day old stationary phase *B. burgdorferi*.

Sample	Gross Conc., mg/L	Residual Viability, %
DE	305.5 ± 24.5	19.8 ± 4.7
NP2	295.4 ± 12.1	54.3 ± 10.4
NP5	332.8 ± 34.7	29.8 ± 7.8
NP7	340.2 ± 14.5	23.4 ± 15.8
DM	308.6 ± 20.2	40.2 ± 9.1
NP2-RP	300.2 ± 8.9	64.5 ± 14.9
Dox., Cefo., Dap. *	22.2; 33.4; 80.1	24.9 ± 7.4

* Abbreviations: conc.—concentration; Dox.—doxycycline; Cefo.—cefoperazone; Dap.—daptomycin.

## Data Availability

Data is contained within the article or Supplementary Material.

## Supplementary Materials

The following are available online at https://www.mdpi.com/article/10.3390/ph15010087/s1, File S1: Statistical comparison of cytotoxic effect of DE and its fractions, File S2: Full NMR analysis.
