# Supplementary material for: Extraction and Fractionation of Bioactives from Dipsacus fullonum L. Leaves and Evaluation of Their Anti-Borrelia Activity
Source: Pharmaceuticals (Basel). 2022 Jan 12;15(1):87. doi: 10.3390/ph15010087 (PMC8779505; doi:10.3390/ph15010087)
Supplement: Supplementary file 1 [file pharmaceuticals-15-00087-s001.zip › pharmaceuticals-1522949-supplementary.pdf]

Supplementary files

Supplementary File S1 – Cytotoxic effect of DE and its fractions

Bonferroni's multiple comparisons test	Mean Diff.	95% CI of diff.	Significant?	Summary
Control vs. DE	79.80	59.69 to 99.91	Yes	****
Control vs. NP2-RP	28.91	10.29 to 47.53	Yes	**
Control vs. NP7	45.33	25.22 to 65.44	Yes	****
Control vs. NP2	36.69	16.58 to 56.80	Yes	***
DE vs. NP2-RP	-50.89	-71.00 to -30.78	Yes	****
DE vs. NP7	-34.46	-55.96 to -12.97	Yes	**
DE vs. NP2	-43.10	-64.60 to -21.61	Yes	***
NP2-RP vs. NP7	16.42	-3.686 to 36.53	No	ns
NP2-RP vs. NP2	7.783	-12.33 to 27.89	No	ns
NP7 vs. NP2	-8.640	-30.14 to 12.86	No	ns

$p < 0.01$ as **, $p < 0.001$ as ***, $p < 0.0001$ as ****

Supplementary File S2 – NMR analysis results for NP2-RP

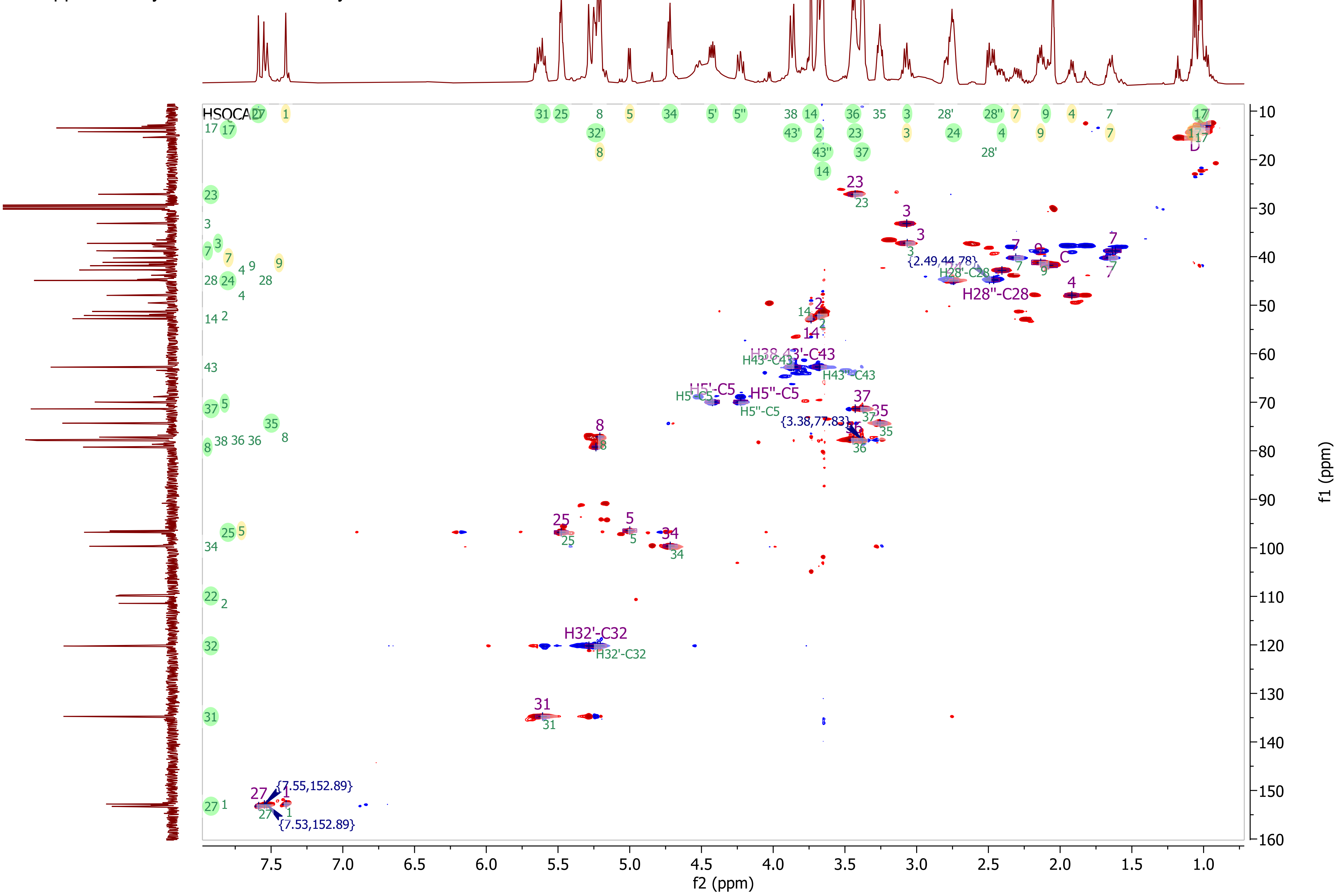

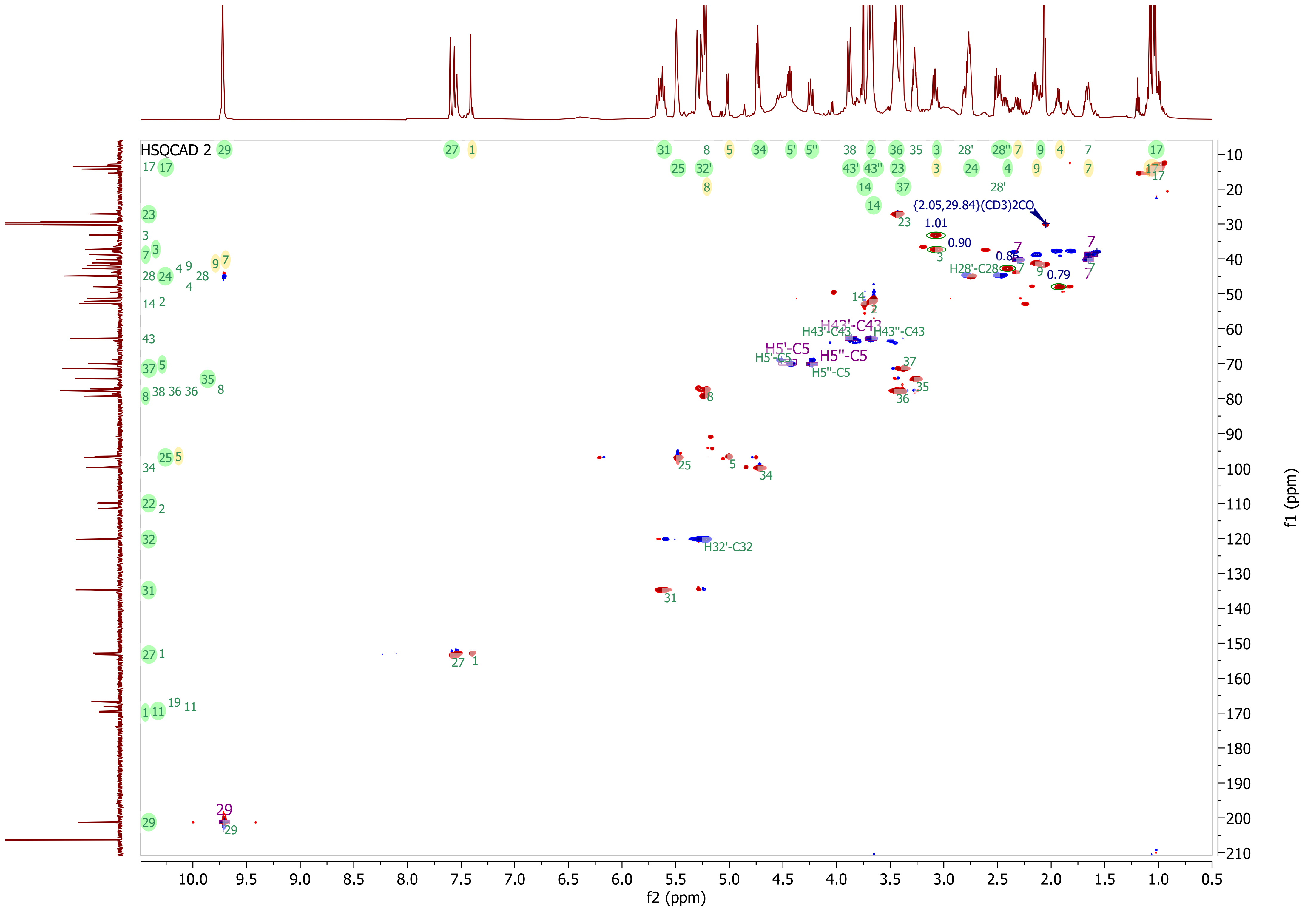

HSQCAD 2
{2.05,29.84}(CD3)2CO
1.01
0.90
0.86
0.79
H28'-C28
H43'-C43
H43"-C43
H5'-C5
H5"-C5
H32'-C32
f2 (ppm)
f1 (ppm)

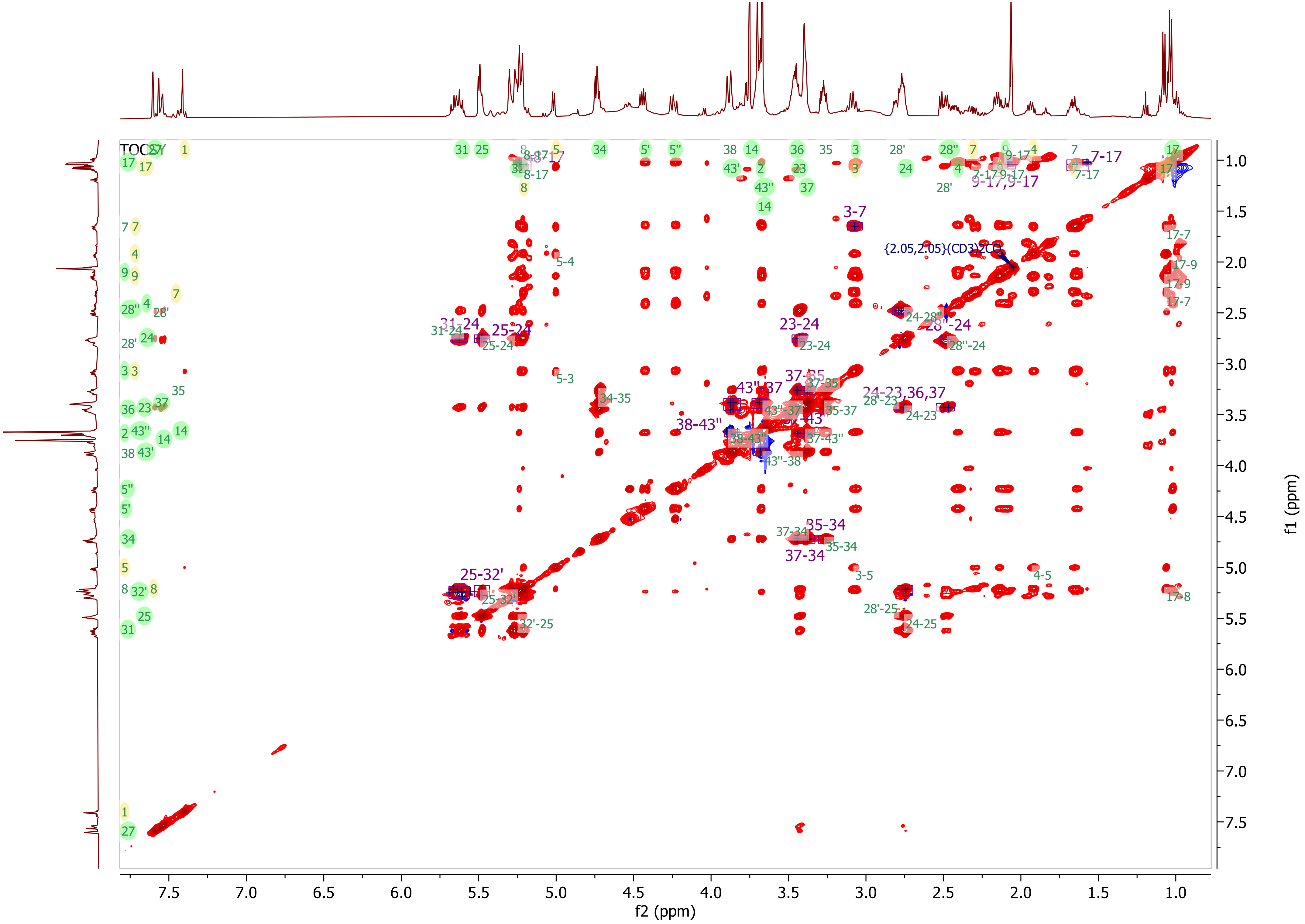

TOCSY
{2.05,2.05}(CD3)2CO
f2 (ppm)
f1 (ppm)
7.5
7.0
6.5
6.0
5.5
5.0
4.5
4.0
3.5
3.0
2.5
2.0
1.5
1.0

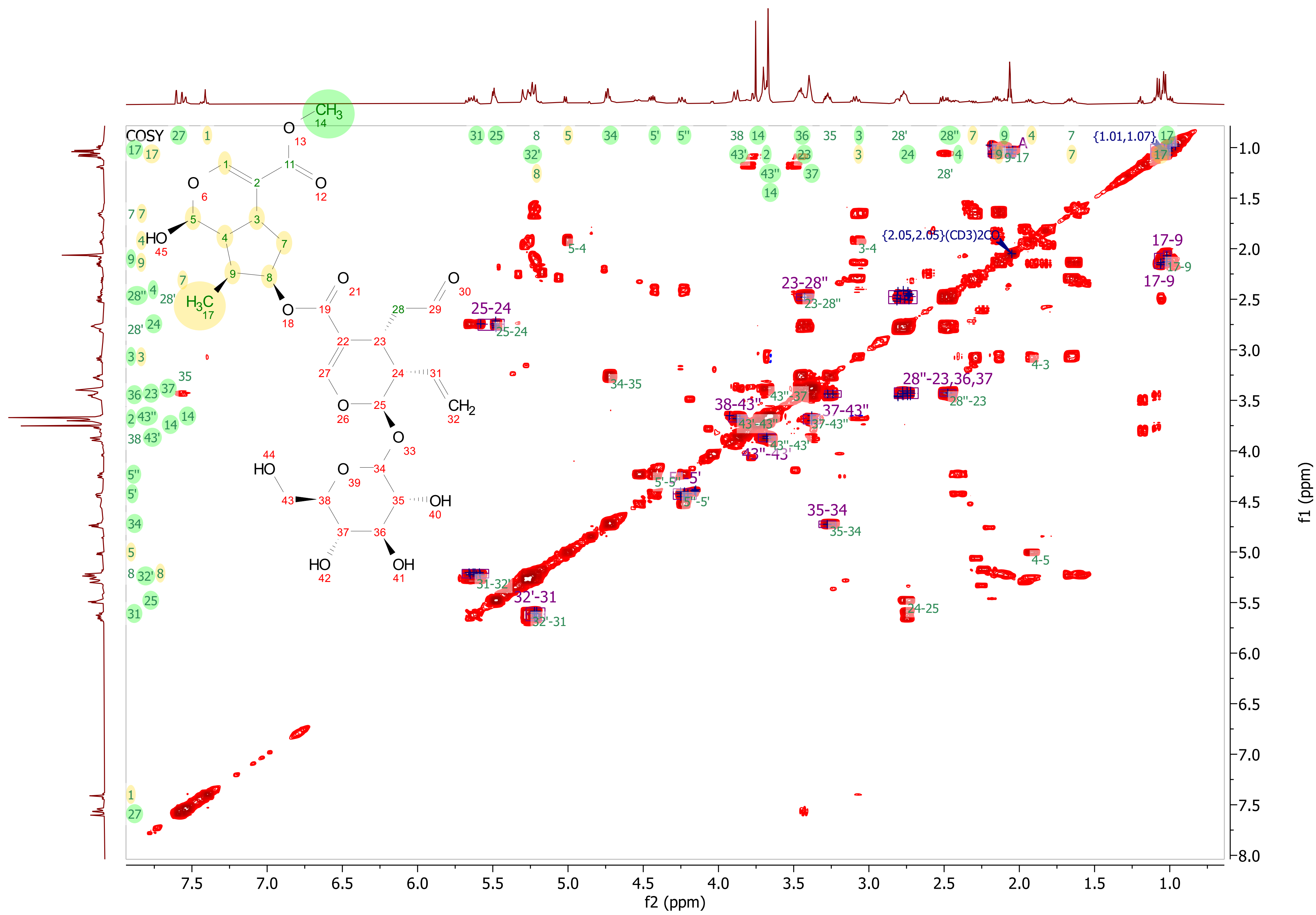
COSY
f2 (ppm)
f1 (ppm)

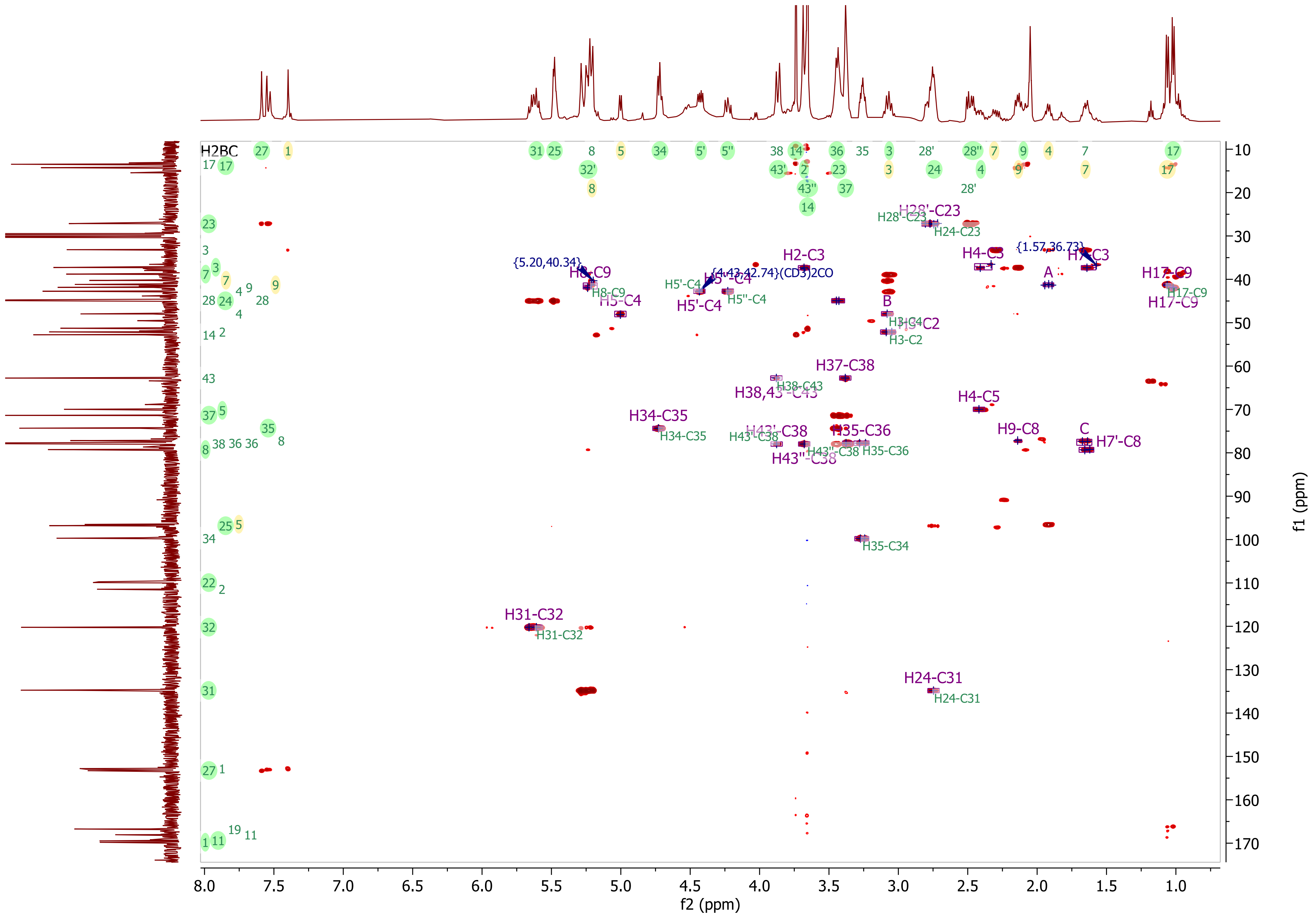
H2BC
H28'-C23
H24-C23
{1.57,36.73}
H7-C3
H2-C3
H4-C3
{5.20,40.34}
H8-C9
{4.43,42.74}(CD3)2CO
H5'-C4
H5"-C4
H5-C4
H17-C9
A
B
H3-C4
H3-C2
H37-C38
H38-C43
H38,43'-C43
H4-C5
H34-C35
H43'-C38
H35-C36
H43"-C38
H35-C36
H9-C8
C
H7'-C8
H35-C34
H31-C32
H24-C31
f2 (ppm)
f1 (ppm)

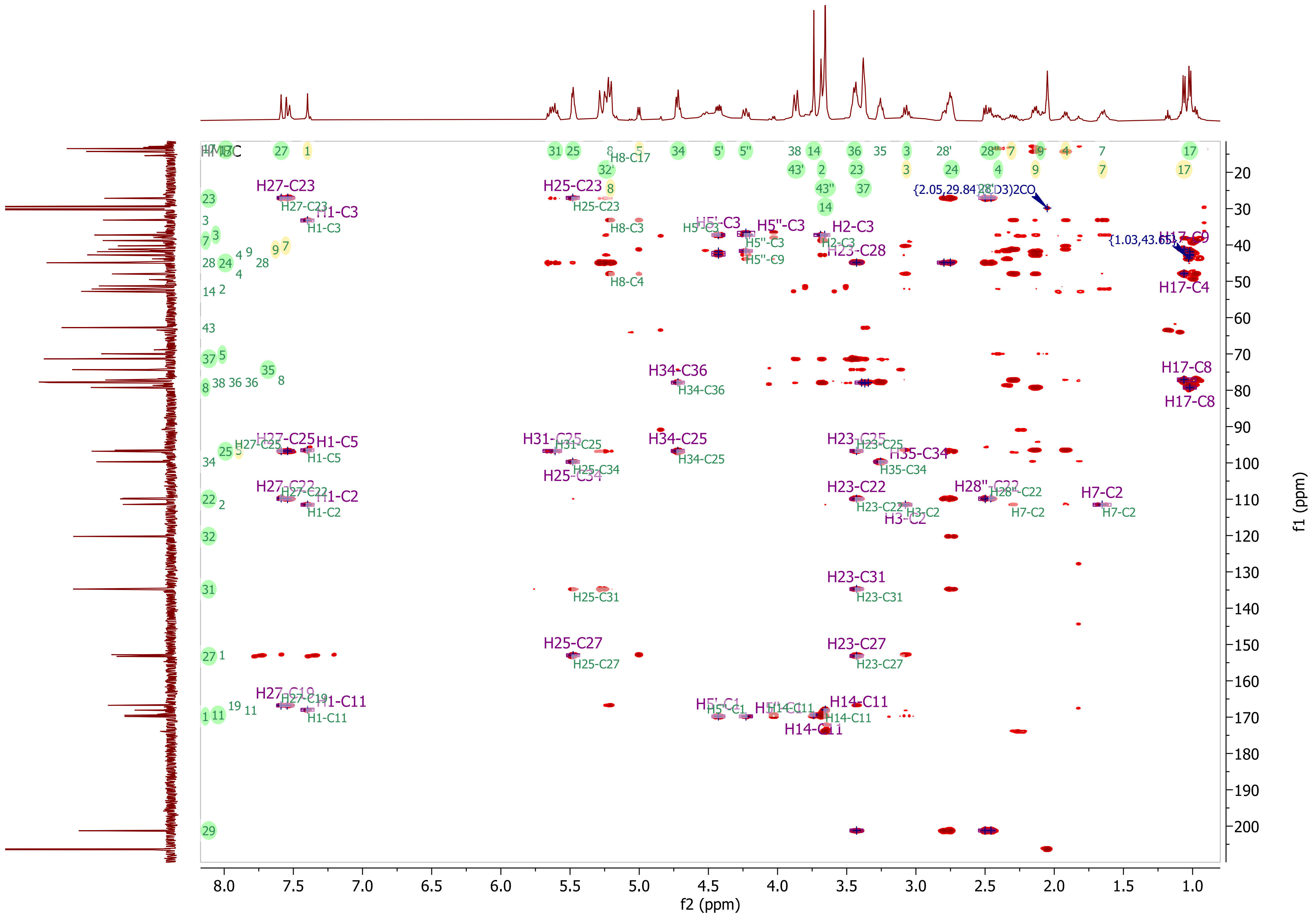

HMBC
H27-C23
H1-C3
H25-C23
H8-C17
H8-C3
H5'-C3
H5"-C3
H2-C3
H23-C28
H5"-C9
H8-C4
H17-C9
H17-C4
{2.05,29.84}(CD3)2CO
{1.03,43.65}
H34-C36
H17-C8
H27-C25
H1-C5
H31-C25
H34-C25
H23-C25
H35-C34
H25-C34
H27-C22
H1-C2
H23-C22
H28"-C22
H7-C2
H3-C2
H23-C31
H25-C31
H25-C27
H23-C27
H27-C19
H1-C11
H5'-C1
H5"-C1
H14-C11
f2 (ppm)
f1 (ppm)
